# Headache-Related Characteristics of Biopsy-Confirmed Giant Cell Arteritis and the Relationship of Transmural Inflammation With Artery Tenderness and Chordal Thickening

**DOI:** 10.7759/cureus.56843

**Published:** 2024-03-24

**Authors:** Sho Shimohama, Noboru Imai, Takuya Tsubata, Kei Shinohara, Asami Moriya, Nobuyasu Yagi, Takashi Konishi, Masahiro Serizawa, Kazuhiro Tashiro

**Affiliations:** 1 Department of Neurology, Japanese Red Cross Shizuoka Hospital, Shizuoka, JPN; 2 Department of Neurology, Keio University School of Medicine, Tokyo, JPN; 3 Headache Center, Japanese Red Cross Shizuoka Hospital, Shizuoka, JPN; 4 Department of Pathology, Japanese Red Cross Shizuoka Hospital, Shizuoka, JPN

**Keywords:** tenderness, biopsy positive, transmural inflammation, headache, giant cell arteritis

## Abstract

Introduction: Giant cell arteritis (GCA) is characterized by headaches, but few studies have examined the detailed characteristics of pathologically confirmed cases. We investigated the characteristics of GCA patients, particularly headache, and their correlation with pathological findings.

Methods: We retrospectively analyzed 26 patients (median age: 77.5 years, male: 38.4%) with GCA who underwent superficial temporal artery (STA) biopsy at the Japanese Red Cross Shizuoka Hospital between May 2001 and February 2022. All patients fulfilled the American College of Rheumatology and European League Against Rheumatism classification criteria for GCA. We focused on the relationship between clinical features, especially headache, and pathological findings.

Results: Twenty-four patients had unilateral, nonpulsatile, intermittent headaches. Transmural inflammation (TMI), a characteristic pathology of GCA, was present in 14 patients. Bivariate analysis revealed significant associations between the TMI and STA-related tenderness (odds ratio [OR]=11, 95% confidence interval [CI]=1.14 to 106.43, p=0.046) and the TMI and STA-related chordal thickening (OR=0.19, 95% CI=0.068 to 0.52, p=0.021).

Conclusions: Headache in GCA patients was often unilateral, nonpulsatile, and intermittent. This study highlights the significant association of TMI with STA tenderness and ligamentous thickening, which has not been reported previously. Abnormal STA findings were significantly associated with pathological changes in GCA patients, emphasizing the importance of these lesions in predicting GCA.

## Introduction

Giant cell arteritis (GCA) is a polysymptomatic disease that affects large- and medium-sized arteries, especially the branches of the proximal aorta, in older adults and causes various symptoms. Headache is one of the most common symptoms of GCA [1]. Headache is typically acute or subacute in onset and may be mild or severe [2]. The current incidence rate of GCA in Japanese patients aged >50 years is 1.48/100,000, which is lower than that in patients from Western countries [3]. To date, very few studies have investigated the clinical features of GCA in Japan [4,5], including one study involving patients without biopsy confirmation [6].

Recently, due to advances in vascular imaging, large-vessel-GCA (LV-GCA), which causes inflammation in large blood vessels other than the temporal artery, has become well known [7,8]. While temporal artery biopsy (TAB) has the disadvantages of invasiveness and lower sensitivity due to "skip lesions", biopsy confirmation may still be the most accurate method for confirming GCA. However, much less is known about the clinical features of GCA, including headaches, only in biopsy-confirmed patients. Hence, LV-GCA is a significant subset of GCA patients, but we focused only on cranial GCA patients with positive pathological findings in this study.

Diagnosing GCA is difficult, and GCA can cause severe cranial neurological symptoms, headache, scalp tenderness, jaw claudication, visual impairment, cerebrovascular accidents, aortic arch syndrome, thoracic aortic aneurysm, and dissection [9]. Therefore, it is clinically important to accurately and early diagnose GCA. We believe it is crucial to determine the clinical features of GCA, including headaches, only in biopsy-confirmed patients to make an early and accurate diagnosis of GCA. To the best of our knowledge, no studies have investigated the characteristics of headaches in East Asian patients with biopsy-positive GCA. Thus, this study aimed to reveal the detailed characteristics of biopsy-positive GCA patients, including headaches, and investigate the relationship between pathological findings and clinical symptoms.

This article was previously posted to the Research Square preprint server on August 18th, 2023.

## Materials and methods

Participants and clinical measurements

**Figure 1:**
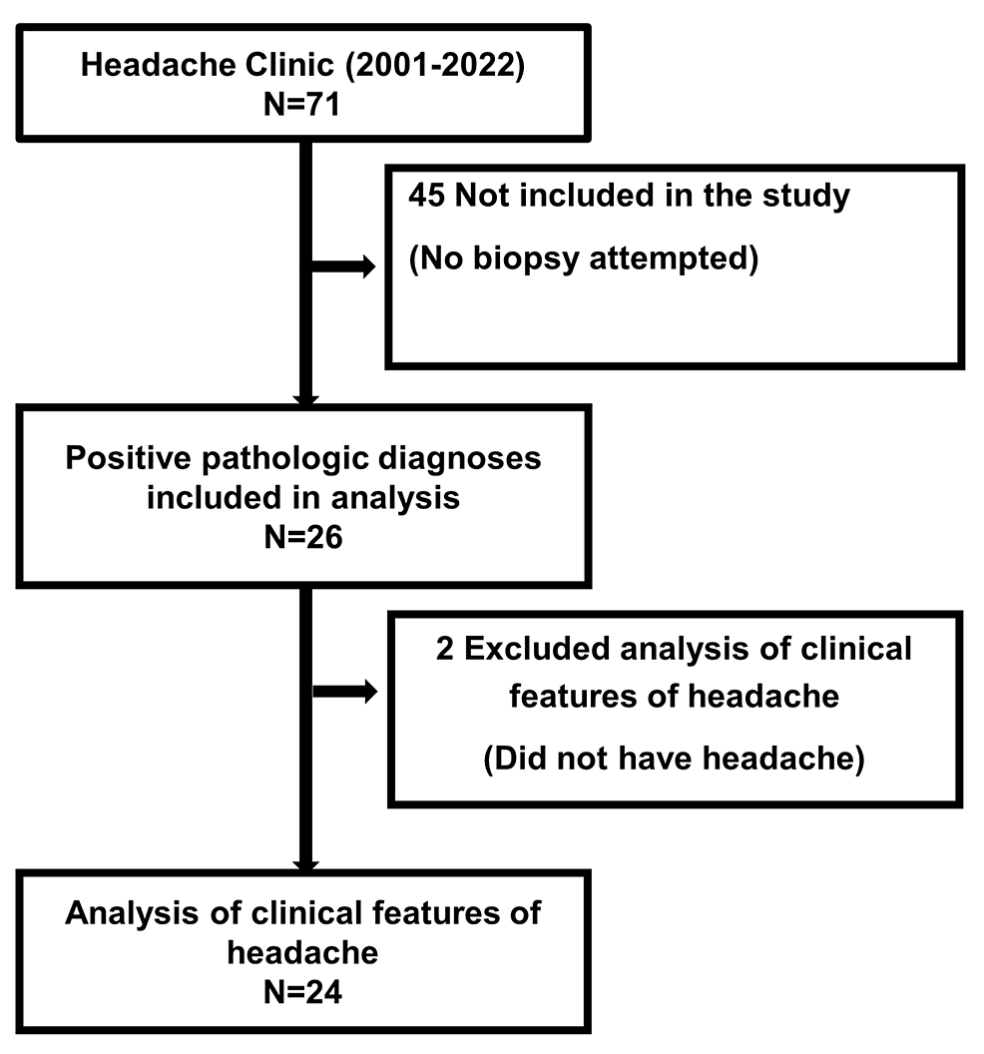
Patient selection flowchart

This retrospective cohort study included patients with a positive pathological diagnosis of GCA who visited Japanese Red Cross Shizuoka Hospital in Shizuoka, Japan, between May 2001 and February 2022 (Figure 1). Japanese Red Cross Shizuoka Hospital is located in Shizuoka City and has a population of approximately 684,900 people. Approximately 94,000 people reside within the medical care zone of the hospital [6]. The following patients were included: patients who met the new classification criteria of the American College of Rheumatology (ACR) [10] and the European League Against Rheumatism (EULAR) announced in 2022 [11]. The following patients were excluded: those who did not meet the pathologically positive classification criteria for ACR1990 [10]. In addition to the 19 patients we reported [6], 71 participants were enrolled (Figure 1). Inflammatory findings from mononuclear-dominant infiltration or granulocytes with polynuclear giant cells, intimal thickening, and destruction of the internal elastic plate were considered the pathologically positive diagnostic criteria of ACR1990 [10]. Twenty-one of the 26 patients (80.8%) had positive pathological diagnoses and were included in the final analysis (Figure 1). Vasculitis, which results in inflammatory cell infiltration from the outer membrane to the media and is called transmural inflammation (TMI), is typical of GCA [12]. The presence of TMI was confirmed in this study, and the characteristics of 24 patients who had headaches during the study course were also examined (Figure 1). Clinical symptoms (headache [bilateral, persistent, pulsating, intensity], jaw claudication, fatigue, fever, weight loss, cranial nerve disorder, polymyalgia rheumatica (PMR), superficial temporal artery (STA) [tenderness, chordal thickening]), treatment, treatment response), laboratory parameters (erythrocyte sedimentation rate [ESR] 1-h value), ultrasound (halo sign), and pathological findings (TMI, giant cells) were collected from the available medical records. A halo sign was considered positive when the wall thickening of the STA was visualized as a low echo around the artery on color Doppler ultrasound.

Standard protocol approval and registration

This study was performed following the principles of the Declaration of Helsinki and the STrengthening the Reporting of OBservational studies in Epidemiology (STROBE) guidelines. The ethics committee of the Japanese Red Cross Shizuoka Hospital (2021-36) approved this study. Given that this study does not involve any active invasion or intervention for research purposes, consent was obtained in accordance with national ethical guidelines (Ethical Guidelines for Medical and Biological Research Involving Human Subjects) using an opt-out approach that provides information about the conduct of the study, including the purpose of the study, and provides an opportunity for refusal whenever possible.

Statistical analysis

Patient characteristics are presented as medians and interquartile ranges for continuous variables and numbers and percentages (%) for categorical variables. We analyzed the relationships between headache characteristics (bilateral, persistent, pulsating, and intense), tenderness and chordal thickening of the STA, the presence of giant cells, and the presence of TMI. Headache intensity was classified into three categories by the numerical rating scale (NRS). An NRS score of 1-3 was considered mild, 4-6 was considered moderate, and 7-10 was considered severe. We evaluated the headache score as the maximum value over the course of time. Bivariate analysis was performed using a two-sided Fisher’s exact test, and statistical significance was set at p < 0.05 (two-sided). Odds ratios (ORs) and confidence intervals (CIs) are also shown for bivariate analyses. JMP version 17 (SAS Institute, Inc., Cary, NC, USA) and IBM SPSS Statistics for Windows, Version 22 (Released 2013; IBM Corp., Armonk, New York, United States) were used for the statistical analyses.

## Results

Clinical characteristics

There were 10 male patients (38.4%), and the median age at disease onset was 77.5 (60-86) years. The mean interval between onset and diagnosis was 37.5 (4-180) days. Twenty-two patients (84.6%) had consulted other medical institutions before visiting our hospital; however, only 6 out of the 22 patients (27.3%) were diagnosed with GCA at these medical institutions. The departments consulted by patients included neurology, rheumatology, and general medicine. The most common manifestation at disease onset was headache in 24/26 patients (92.3%), and the peak ESR was 88.2 ± 28.6 (mm/h). Sixteen of the 21 patients (76.2%) had a halo sign on US. All patients were treated with prednisolone (PSL); no other immunosuppressive drugs were used. All patients demonstrated improvement with initial steroid treatment. However, five patients (19.2%) relapsed during steroid treatment. Ocular manifestations were reported in five patients (19.2%). Six patients (23.1%) had general fatigue, eight (30.8%) had a fever, and eight (30.8%) had weight loss. PMR was observed in five patients (19.2%). Jaw claudication was observed in 11 patients (42.3%). Furthermore, 18 patients (69.2%) had STA tenderness, and 22 (84.6%) had chordal thickening of the STA.

Clinical characteristics of headache

The headache characteristics of 24 patients with headaches during the study course were investigated. We examined headache characteristics in detail (Table 1). The most common location was the temporal region, as reported in 17 patients (70.8%). Bilateral involvement was observed in 11 patients (46%), whereas unilateral involvement was observed in 13 (54.2%). The headache pulsated in nine patients (37.5%), nonpulsated in 15 (62.5%), persistent in 9 (37.5%), and intermittent in 15 (62.5%). Headache intensity was classified as follows: severe (nine patients [37.5%]), mild-to-severe (six patients [25%]), or mild (nine patients [37.5%]).

**Table 1:** Demographic and clinical characteristics of headache patients (n=24).

Characteristics	Number of subjects	%
Place		
temporal area	17	70.8
occipital area	3	12.5
temporal and parietal area	2	8.3
temporal and occipital area	1	4.2
parietal area	1	4.2
Unilateral	13	54.2
Pulsating	9	37.5
Intensity		
severe	9	37.5
mild-to-severe	6	25.0
mild	9	37.5
Persistent	9	37.5

Pathological findings

Mononuclear-dominant infiltration was observed in all patients. Granulocytes with polynuclear giant cells were observed in 21/26 patients (80.8%). Intimal thickening was observed in all patients. Destruction of the internal elastic plate was observed in 19/26 patients (73.1%). The TMI represents inflammatory cell infiltration from the adventitia to the media and is a typical pathological finding in GCA [7]. TMI was observed in 14/21 patients (66.7%). Tables 2, 3 show the relationships between the presence of giant cells and headache and between the TMI and headache. There was no significant association between the characteristics of headache (bilateral, persistent, pulsating, or intense) and the presence of giant cells (OR = 2.06, 95% CI 0.28 to 15.36, p = 0.63; OR = 1.14, 95% CI 0.15 to 8.59, p = 1.00; OR = 1.5, 95% CI 1.049 to 2.15, p = 0.12; and OR = 1.87, 95% CI 0.24 to 14.65, p = 0.61, respectively) or between headache and the TMI (OR = 0.17, 95% CI 0.015 to 1.90, p = 0.18; OR = 0.80, 95% CI 0.10 to 6.10, p = 1.00; OR = 4.29, 95% CI 0.39 to 47.62, p = 0.33; and OR = 0.45, 95% CI 0.039 to 5.21, p = 1.00, respectively). Moreover, there was no significant association between STAs (temporal artery tenderness, chordal thickening, or halo signs) and the presence of giant cells (OR = 2.5, 95% CI 0.31 to 20.45, p = 0.57; OR = 12, 95% CI 0.81 to 177.44, p = 0.10; and OR = 2.5, 95% CI 0.17 to 37.26, p = 0.49, respectively). No significant association was observed between the halo sign and the TMI (OR = 2.4, 95% CI 0.12 to 46.39; p = 1.00). However, bivariate analysis revealed a significant association between the TMI and STA-related tenderness (OR = 11, 95% CI 1.14 to 106.43; p = 0.046) and between the TMI and STA-related chordal thickening (OR = 0.19, 95% CI 0.068 to 0.52; p = 0.021).

**Table 2:** Relationship between clinical features, including headache, and the presence of giant cells. *The relationship between clinical features, including headache, and the presence of giant cells was assessed using bivariate analysis with Fisher's exact test. p <0.05 was considered to indicate statistical significance (two-sided). Odds ratios (ORs) and 95% confidence intervals (CIs) are also shown for bivariate analyses. OR: Odds ratio; CI: confidence interval

Clinical Feature		Giant cells		*p-value	*OR (95%CI)
		positive	negative		
Bilateral	unilateral	11	2	0.63	2.06 (0.28 to 15.36)
	bilateral	8	3		
Persistent	persistent	7	2	1.00	1.14 (0.15 to 8.59)
	intermittent	12	3		
Pulsating	pulsating	9	0	0.12	1.5 (1.049 to 2.15)
	nonpulsating	10	5		
Intensity	moderate/severe	14	3	0.61	1.87 (0.24 to 14.65)
	mild	5	2		
Tenderness of superficial temporal artery	positive	15	3	0.57	2.5 (0.31 to 20.45)
	negative	4	2		
Cord thickening of superficial temporal artery	positive	18	3	0.10	12 (0.81 to 177.44)
	negative	1	2		
Halo sign (n=21)	positive	15	2	0.49	2.5 (0.17 to 37.26)
	negative	3	1		

**Table 3:** Relationships between clinical features, including headache, and TMI. *Relationships between clinical features, including headache, and the TMI were assessed using bivariate analysis with Fisher's exact test. p<0.05 was considered to indicate statistical significance (two-sided). Odds ratios (ORs) and 95% confidence intervals (CIs) are also shown for bivariate analyses. TMI: Transmural inflammation; OR: odds ratio; CI: confidence interval

Clinical Feature		TMI		*p-value	*OR (95%CI)
		Positive	Negative		
Bilateral	unilateral	6	5	0.18	0.17 (0.015 to 1.90)
	bilateral	7	1		
Persistent	persistent	5	2	1.00	0.80 (0.10 to 6.10)
	intermittent	8	4		
Pulsating	pulsating	6	1	0.33	4.29 (0.39 to 47.62)
	nonpulsating	7	5		
Intensity	moderate/severe	9	5	1.00	0.45 (0.039 to 5.21)
	mild	4	1		
Tenderness of superficial temporal artery	positive	11	2	0.05	11 (1.14 to 106.43)
	negative	2	4		
Cord thickening of superficial temporal artery	positive	13	3	0.02	0.19 (0.068 to 0.52)
	negative	0	3		
Halo sign (n=21)	positive	12	5	1.00	2.4 (0.12 to 46.39)

Treatment

In all the patients, steroid treatment was administered after biopsy or ultrasound. All patients were treated with PSL; three (11.5%) patients were treated with <40 mg/day, whereas 23 (88.5%) were treated with 40-60 mg/day. Three patients were treated with intravenous high-dose methylprednisolone (1000 mg, 3 days) before PSL therapy. Recurrence was observed in five patients, and treatment responsiveness at recurrence was good in all patients. There was no significant association between the presence of giant cells and treatment response (OR = 0.25: 95% CI 0.029 to 2.18; p = 0.24). Moreover, there was no significant association between the TMI and treatment response (OR = 0.22: 95% CI 0.027 to 1.85; p = 0.28).

## Discussion

This study investigated the clinical features and characteristics of headache in patients with a positive pathological diagnosis of GCA. Notably, few reports on GCA have detailed the characteristics of headaches in patients with positive pathological findings. In this study, 24 patients had unilateral, nonpulsatile, and intermittent headaches. The TMI has been identified as a major pathological feature of GCA. However, its association with the clinical features of GCA patients has not been fully elucidated. This study demonstrated the significant associations of the TMI with STA tenderness and chordal thickening.

Headache is a critical discriminating symptom [2,7]. GCA-induced headaches are typically observed as unilateral, pulsatile, or persistent [13]. In contrast, the characteristics of GCA-induced headaches in this study tended to be unilateral, nonpulsatile, or intermittent. This result may indicate GCA-induced headaches in Japanese patients. The ischemic symptoms of GCA are associated with intimal hyperplasia [14]. All patients in this study had vascular lumen stenosis, regardless of the pulsatile nature of the headache. It has been reported that GCA headaches are often pulsatile, reflecting vascular ischemia [13], although to the best of our knowledge no detailed data are available. In this study, 62.5% of the patients were nonpulsating, which may be a characteristic of GCA headaches in Japanese patients. Cranial GCA is well known to be associated with clinical symptoms reflecting pathological conditions. Therefore, we focused on the relationship between the presence of giant cells and the characteristics of headache, although the difference was not significant.

In this study, all patients had pathological findings. We focused on TMI findings and the characteristics of GCA patients and found that the TMI was significantly associated with STA tenderness and chordal thickening. STA tenderness and chordal thickening are the major clinical features of GCA. A previous meta-analysis reported a sensitivity and specificity of 36% and 81.4%, respectively, for STA tenderness and 44.4% and 90.6%, respectively, for chordal thickening [15]. The TMI represents inflammatory cell infiltration from the adventitia to the media and is a typical pathological finding in GCA [12]. A previous study reported that TMI was present in 77.5% (n = 354) of patients, and multinucleated giant cells were found in 74% of TMI patients [12]. This study revealed the TMI in 14/21 patients (66.7%). Additionally, giant cells were found in all the TMI patients. The significant associations of the TMI with STA tenderness and chordal thickening indicated arterial inflammation. We also investigated the association between pathological findings and treatment outcome, but there was no significant association between the presence of giant cells or the TMI and treatment response. A previous study also revealed no correlation between histology and treatment outcome [16].

Compared with the results mentioned in previous retrospective studies, this study reports differences in the clinical features of GCA patients with a positive pathological diagnosis in Japan (Table 4) [4,5]. The average age of onset in our study was 70 years. Headaches occurred in approximately 90% of our patients, which is greater than what has been reported in other studies. In our study, neurologists treated many patients; hence, headaches may have been a common symptom in our cohort. PMR complications were common in previous studies. However, both this study and previous ones reported a high rate of abnormal findings in the STA. Only our study investigated the halo sign. The halo sign is a representative ultrasound finding of GCA [17], and previous studies have reported a high specificity of 96% for GCA [18]. In this study, 16/21 patients (76.2%) exhibited a positive halo sign. It was suggested that the presence of a halo sign on ultrasound may also assist in diagnosing GCA. It is considered important even in the new classification criteria of the ACR and EULAR [11], where five points are considered if positive pathological or ultrasound findings are present. Although one retrospective study investigated Chinese patients with GCA, few studies have reported the clinical characteristics of GCA [19]. A total of 70 patients were diagnosed with GCA; TAB was performed in 42 (60.0%) patients, and 32/42 (71.4%) patients had positive pathological findings of GCA [19]. Forty-eight (68.6%) patients had headache, and 17 (24.3%) had abnormal temporal artery findings [19].

**Table 4:** Past reports of pathologically positive GCA patients in Japan. GCA: Giant cell arteritis; N/A: not applicable

Cases	Iesaka	Oiwa	Our cases
n	11	16	26
Age	71	76	77
Female/male	1(9%)	6/15(40%)	10(38%)
Headache	4(36%)	12(75%)	24(92%)
Fever	7(64%)	12(75%)	8(31%)
Weight loss	N/A	10/15(67%)	8(31%)
Polymyalgia rheumatica	5(45%)	3(19%)	5(19%)
Cranial nerve symptoms	2(18%)	1(6%)	5(19%)
Halo sign	N/A	N/A	17/21(81%)
Thickening of the superficial temporal artery	3(27%)	14(88%)	22(85%)
Superficial temporal artery tenderness	3(27%)	8/15(53%)	18(69%)

GCA diagnosis can be challenging. In this study, the mean days from the time of initial diagnosis to the time of diagnosis are 38 (4-180). If the diagnosis is delayed, the time for appropriate treatment will be missed, leading to serious symptoms such as vision loss. STA inflammation, including STA tenderness and chordal thickening, is diagnostic for GCA, although multiple diseases other than GCA cause inflammation of the STA. For example, necrotizing vasculitis, immunoglobulin G4-related disease, sarcoidosis, and varicella zoster virus vasculitis also cause inflammation of the STA [20]. TAB is the gold standard for diagnosis, and vascular imaging (ultrasound, computed tomography, magnetic resonance imaging, and positron emission tomography) is also useful for diagnosis. Although there have been few reports of pathologically positive GCA, this study is clinically important because it is the first to investigate in detail the characteristics of pathologically positive GCA in East Asia.

This study has several limitations. First, this was a single-center, retrospective study. Second, because of our limited access to ultrasound medical records, 21 patients had halo signs and TMI findings. Third, the bivariate analysis results should be interpreted considering the sample size. Since GCA is rare in East Asia, it was difficult to examine many patients, and we believe that the examination of 26 patients is important.

## Conclusions

To the best of our knowledge, this study is the first to examine the clinical features of GCA, including headaches, in East Asian patients with GCA with only positive pathological findings. Notably, more patients presented with headache symptoms in this study than in previous studies, and the headaches presented as unilateral, nonpulsatile, or intermittent. Furthermore, the TMI was significantly associated with STA tenderness and chordal thickening. Timely diagnosis of GCA based on understanding various characteristics followed by appropriate treatment is clinically important for preventing serious symptoms such as vision loss. We showed that abnormal STA findings were significantly related to GCA pathological findings; thus, it is necessary to pay attention to abnormal STA findings when suspecting GCA.
